# Identification and expression characteristics of porcine embryonic circRNAs during the peri-implantation

**DOI:** 10.1186/s12864-026-12713-y

**Published:** 2026-03-05

**Authors:** Shengchen Gu, Chen Zhou, Lei Jiang, Shifei Lin, Yongzhong Wang, Xinxiu Li, Junying Ai, Ting Gu, Zicong Li, Gengyuan Cai, Zhenfang Wu, Linjun Hong

**Affiliations:** 1 State Key Laboratory of Swine and Poultry Breeding Industry, National Engineering Research Center for Breeding Swine Industry, College of Animal Science, South China Agricultural University, Guangzhou, 510642 China; 2 Guangdong Provincial Key Laboratory of Agro-Animal Genomics and Molecular Breeding, South China Agricultural University, Guangzhou, 510642 China; 3 College of Animal Science and Technology, Luoyang Key Laboratory of Animal Genetic and Breeding, Henan University of Science and Technology, Luoyang, 471003 China; 4 Yunfu Subcenter of Guangdong Laboratory for Lingnan Modern Agriculture, Yunfu, 527300 China; 5 National Regional Gene Bank of Livestock and Poultry (Gene Bank of Guangdong Livestock and Poultry), Guangzhou, 510642 China

**Keywords:** circRNA, Pig, Conceptus, Peri-implantation, Development

## Abstract

**Background:**

One of the essential periods of embryonic development and morphological transformation in pigs is the peri-implantation period, occurring on gestation days (D) 9, D12, and D15. circular RNAs (circRNAs), a non-coding RNA (ncRNA) family member, are suspected to play key roles in numerous biological processes. However, the role of circRNAs during early pregnancy in pigs has not yet been elucidated.

**Results:**

To address this, we integrated RNA-seq, bioinformatics analyses, and molecular biology experiments to define the circRNA expression landscape in embryos at gestational D9, D12, and D15. A total of 6,931 circRNAs were identified, with 183, 634, and 82 differentially expressed circRNAs (DEcircRNAs) found in the differential expression analyses of the three sample pairs (D9 vs. D12, D9 vs. D15, and D12 vs. D15). Functional enrichment analysis revealed significant associations with multiple biological processes and pathways relevant to embryonic development. The intersection of these differential expression analyses yielded seven hub circRNAs. By integrating porcine miRNAs from miRBase with mRNA sequencing data, a competing endogenous RNA (ceRNA) network comprising two circRNAs, seven mRNAs, and 166 miRNAs was constructed, suggesting a potential role for circRNAs as molecular sponges that may sequester miRNAs and thereby influence mRNA expression and related biological processes. To validate the back-spliced circular structure of the circRNAs, polymerase chain reaction (PCR) amplification and Sanger sequencing were performed. Additionally, three circRNAs were randomly selected for real-time quantitative PCR (RT-qPCR) validation, and their relative expression levels were consistent with sequencing results.

**Conclusions:**

This study not only characterized the circRNA expression profile of pig embryos on D9, D12, and D15 of pregnancy but also established a solid foundation for further exploring the molecular mechanisms of key circRNAs in pig embryo development, thereby providing insights into the processes of implantation and embryo survival.

**Supplementary Information:**

The online version contains supplementary material available at 10.1186/s12864-026-12713-y.

## Introduction

Porcine embryonic development and implantation during early pregnancy are highly complex biological processes. Successful conceptus extension during the peri-implantation period is critical for subsequent embryo development and survival, as inadequate conceptus extension results in approximately 20% embryo loss and directly affects within-litter birth-weight variation and survival of piglets after birth [1, 2]. Porcine embryos begin to develop after successful fertilisation to form a zygote, which develops to the morula stage around the 60th to 72nd hour after fertilisation, and the embryo enters the uterus and develops to the blastocyst stage around D5 of gestation [3]. During early gestation, the porcine blastocyst rapidly transitions from a spherical to a tubular form after hatching from the zona pellucida on D7 [4]. By D12, the embryo elongates rapidly, reaching up to 1 m in length [5, 6]. At D13, it begins to attach to the uterine luminal epithelium [3, 7]. After attachment, the filamentous conceptus continues to elongate and can exceed 1 m by D16 [8]. Implantation is completed between D18 and D24 [9]. During embryonic elongation, extensive changes in conceptus and endometrial gene expression are essential for establishing pregnancy and creating a supportive uterine environment [1, 10]. Therefore, elucidating the molecular mechanisms underlying porcine embryo elongation is critical for improving pregnancy success. Understanding these developmental events requires a detailed analysis of the underlying regulatory molecules. In this context, circRNAs have recently emerged as a class of RNA molecules and have been increasingly investigated in diverse developmental and physiological processes [11, 12]. Among them, these molecules form a covalently closed-loop structure through back-splicing [13]. circRNAs have been shown to regulate gene expression. For instance, ciRS-7 functions as a molecular sponge for miR-7, leading to the suppression of miR-7 activity and the consequent upregulation of its target mRNAs [14]. Several studies have found that circRNAs exist in the brain [15], muscle [16], testis [17], uterus [9], and other tissues, and have investigated their potential biological functions. It was found that circINHA up-regulates CTGF, promoting proliferation and inhibiting apoptosis of porcine granulosa cells by competitively binding to miR-10a-5p [18]. Previous studies have shown that circBTBD7 promotes the growth of immature porcine support cells by inactivating the p38-MAPK signalling pathway [19]. Previous studies found that circRNAs can be dynamically expressed and interact with muscle-related genes by means of ceRNAs, suggesting that they play an important role in the development of skeletal muscle in porcine embryos [20, 21]. Evidence from previous work indicates that circRNAs are abundantly present and temporally as well as spatially regulated in the porcine fetal brain, highlighting their significant contribution to mammalian brain development [15]. Previous work, including our own, has shown abundant circRNA expression in the porcine endometrium on day 12 of gestation, with breed-specific patterns that may influence embryo survival [9]. Beyond endometrial regulation, our earlier transcriptomic data from conceptuses at D9, D12, and D15 demonstrated extensive peri-implantation gene reprogramming, reflecting coordinated maternal–conceptus molecular dynamics [22]. Accordingly, this study investigates embryo circRNA expression to further elucidate regulatory mechanisms driving conceptus development. However, despite these reported functional circRNAs, recent evolutionary analyses have questioned the biological relevance of most circRNAs. A comparative study showed that back-splicing is rare, largely non-conserved, and likely reflects splicing noise, with over 97% of circRNAs estimated to be non-functional [23, 24]. These observations underscore the need for caution when interpreting circRNA expression patterns. Collectively, these findings indicate that circRNAs are broadly present in pigs and could participate in the regulation of embryonic development during gestation. Nevertheless, their expression profiles and underlying regulatory roles in porcine embryos at the early stages of pregnancy remain unclear.

To summarise, this study focuses on examining the expression dynamics of circRNAs in early pregnancy porcine embryos and their relationship with embryonic development. By identifying the pathways concerned by these circRNAs in embryonic development and the relationship with other RNAs, we aim to shed new light on the molecular processes that drive embryonic development.

## Materials and methods

### Experimental animals and sample collection

Healthy Yorkshire sows with comparable age and genetic background were obtained from Guangdong WENS Food Group Co., Ltd. (Yunfu, China) and managed under identical conditions. Estrus synchronization, artificial insemination, and sample collection were performed as previously described [22]. Briefly, nine second-parity sows were inseminated at estrus onset (D0), followed by a second insemination 12 h later. Sows were randomly assigned to gestational D9, D12, or D15 (*n* = 3 per group). For each gestational stage, embryos collected from one sow were pooled and considered one biological replicate. Sample identifiers within each developmental stage represent independent biological replicates and do not indicate matched samples across stages.

### RNA extraction, library preparation, and RNA sequencing

Total RNA extraction, library preparation, and RNA sequencing were conducted using the same samples and protocols as described in our previously published study [22]. Briefly, total RNA was extracted from embryonic samples, quality assessed, and used for rRNA-depleted library construction. Sequencing was performed on an Illumina HiSeq platform to generate 150 bp paired-end reads. The raw sequencing data used in this study are identical to those previously reported in our earlier study [22]; however, the downstream analytical strategies and biological questions addressed here are distinct.

### Quality control and mapping

Raw RNA-seq data from nine datasets originally published in [22] were re-analyzed in the present study.

Compared with the original analysis, reads were aligned to the updated Sus scrofa reference genome (Sscrofa11.1). To obtain high-quality reads, raw reads were processed with Trimmomatic (v0.30) to remove adapters, PCR primers, and low-quality bases. Illumina TruSeq adapters were removed using the TruSeq3-SE.fa adapter library. Low-quality bases were further trimmed with a sliding window of 4 bp, requiring an average quality of at least 20, and bases with quality below three at the 5′ or 3′ ends were removed. Reads shorter than 36 bp after trimming were discarded. Subsequent analyses were performed using these clean reads. The Sus scrofa reference genome (Pig Sscrofa11.1) and annotation files (Ensembl release 115) were downloaded for read alignment and gene annotation. The use of the updated genome assembly and annotation enabled a refined identification of both mRNAs and lncRNAs, particularly during the peri-implantation stage.

### circRNA identification, quantification, and host gene assignment

To enable circRNA identification, clean reads were first mapped to the indexed porcine reference genome using BWA-MEM (v0.7.12-r1039) with default settings, employing eight threads for parallel processing. circRNAs were identified from the alignment files using CIRI2 (v2.0) with the *Sus scrofa* 11.1 reference genome and its annotation files. A minimum Back-Spliced Junction (BSJ) read support of ≥ 2 reads per sample was required, and only circRNAs detected in at least two samples were considered high-confidence candidates. Repetitive sequences and reads mapping to multiple genomic locations were filtered according to the default CIRI2 settings. Expression levels of circRNAs were quantified based on BSJ reads and normalized using SRPBM (spliced reads per billion mapping), calculated as follows:

$$SRPBM=\frac{Ri\times{10}^{9}}{Rtotal}$$

In this formula, Ri is the number of BSJ reads for a circRNA, and Rtotal is the total number of mapped reads [25].

To assign host genes, BSJ coordinates were mapped to Ensembl gene annotations: Each circRNA was then assigned to a host gene if its BSJ overlapped annotated exons; BSJ overlapping multiple genes were labeled as ambiguous, and those outside any annotated gene were considered intergenic.

### Differential expression and hub circRNA analyses

Differential expression analysis was performed using DESeq2 (v1.42.1) with a negative binomial model, based on the BSJ read counts of circRNAs. *P-values* were adjusted with the Benjamini–Hochberg method, and circRNAs with adjusted *p-value* (FDR) < 0.05 and |log2FoldChange| >1 were defined as significantly differentially expressed. circRNAs that are differentially expressed across all three groups are defined as hub circRNAs.

### mRNA quantification and hub mRNA Identification

For gene expression profiling, the clean reads were independently aligned to the reference genome (here sscrofa11.1 contrary to sscrofa10.2 used in [22]) using HISAT2 (v2.2.1). Transcript assembly was performed with StringTie (v3.0.0), and transcripts from all biological replicates within each group were merged using Cuffmerge (v2.2.1) to obtain a unified transcript set. Transcript sequences were extracted with Gffread (v0.12.7). Differential expression analysis across the three developmental stages was conducted using DESeq2 (v1.42.1), and transcript abundance was expressed as fragments per kilobase of exon per million mapped fragments (FPKM). Genes with |log2FoldChange| > 1 and a FDR < 0.05 were considered significantly differentially expressed. Hub mRNAs were determined by taking the intersection of differentially expressed genes from all pairwise stage comparisons.

### GO and KEGG enrichment analysis of host genes of DEcircRNAs

Functional enrichment analyses were conducted on the host genes of DEcircRNAs to provide contextual information, rather than to directly infer circRNA functions, as the functions of circRNAs may differ from those of their host genes. To ensure reliable gene-based enrichment, only exonic circRNAs with unambiguous host gene annotation were retained for Gene Ontology (GO; http://www.geneontology.org/) and Kyoto Encyclopedia of Genes and Genomes (KEGG; http://www.genome.jp/kegg/) analyses. Intronic and intergenic circRNAs, as well as those that could not be confidently assigned to specific host genes, were excluded. For GO analysis, genes were assigned to GO terms to obtain functional classifications and counts. Hypergeometric testing was then applied to identify significantly enriched terms against the background gene set. GOseq (v1.50.0) was used to perform GO enrichment analysis while correcting for gene length bias. For pathway analysis, host genes were mapped to the KEGG, which links genomic information with higher-level functional networks. Enrichment of KEGG pathways was assessed using KOBAS (v3.0).

### Analysis of hub mRNAs and hub circRNAs and construction of the ceRNA network

For ceRNA network construction, circRNAs that showed cross-sample consistency and robust expression across D9, D12, and D15 were first retained, regardless of their genomic category. To characterize their temporal expression patterns, hierarchical clustering heatmaps were generated for hub circRNAs and hub mRNAs, respectively. circRNAs and mRNAs that exhibited similar temporal expression trends were subsequently selected for ceRNA network construction. To predict potential miRNA-mediated interactions, 457 known porcine miRNAs were retrieved from miRBase (https://www.mirbase.org/). circRNA and mRNA target prediction was performed based on their reconstructed sequences. Exonic circRNAs were reconstructed by concatenating circularized exons rather than using the full genomic interval. Exon–intron circRNAs (EIciRNAs) were assembled according to the exact exon–intron architecture defined by their BSJ, ensuring that only intronic regions truly retained in the circular RNA were included. For intergenic circRNAs, the BSJ-flanked intergenic sequences were extracted and used as the circular RNA sequences. For mRNAs, the 3’-untranslated region (3’ UTR) sequences were used as the basis for miRNA target prediction. miRNAs interacting with both circRNAs and mRNAs were then predicted using two widely used tools, miRanda (v3.3a) and TargetScan (v7.0). For miRanda, parameters were set as score ≥ 150, energy threshold < -30, and strict 5′ seed pairing was applied. TargetScan was run with default parameters. The resulting interactions were integrated and visualized as a ceRNA network using Cytoscape (v3.8.2).

### Verification of circRNA circularisation by PCR and Sanger sequencing

Embryonic total RNA was isolated using the Trizol method and reverse-transcribed into cDNA with the PrimeScript™ RT reagent Kit and gDNA Eraser (Takara, Liaoning, China). Divergent primers (Supplementary Material 1) were applied for PCR amplification, and the products were validated by Sanger sequencing to confirm circRNA BSJ.

### Verification of circRNA relative expression levels

To validate circRNA sequencing results, RT-qPCR was conducted to assess the relative expression of embryonic circRNAs at gestation D9, D12, and D15. Divergent primers (Supplementary Material 2) and the PowerUp SYBR Green Master Mix (Thermo Fisher Scientific, Vilnius, Lithuania) were used, with GAPDH serving as the internal control. Data were processed in Excel, and relative expression levels were calculated using the 2^−ΔΔCt^ method. Group differences were evaluated with independent-sample t-tests.

## Results

### Morphological changes of porcine embryos during early pregnancy

**Fig. 1:**
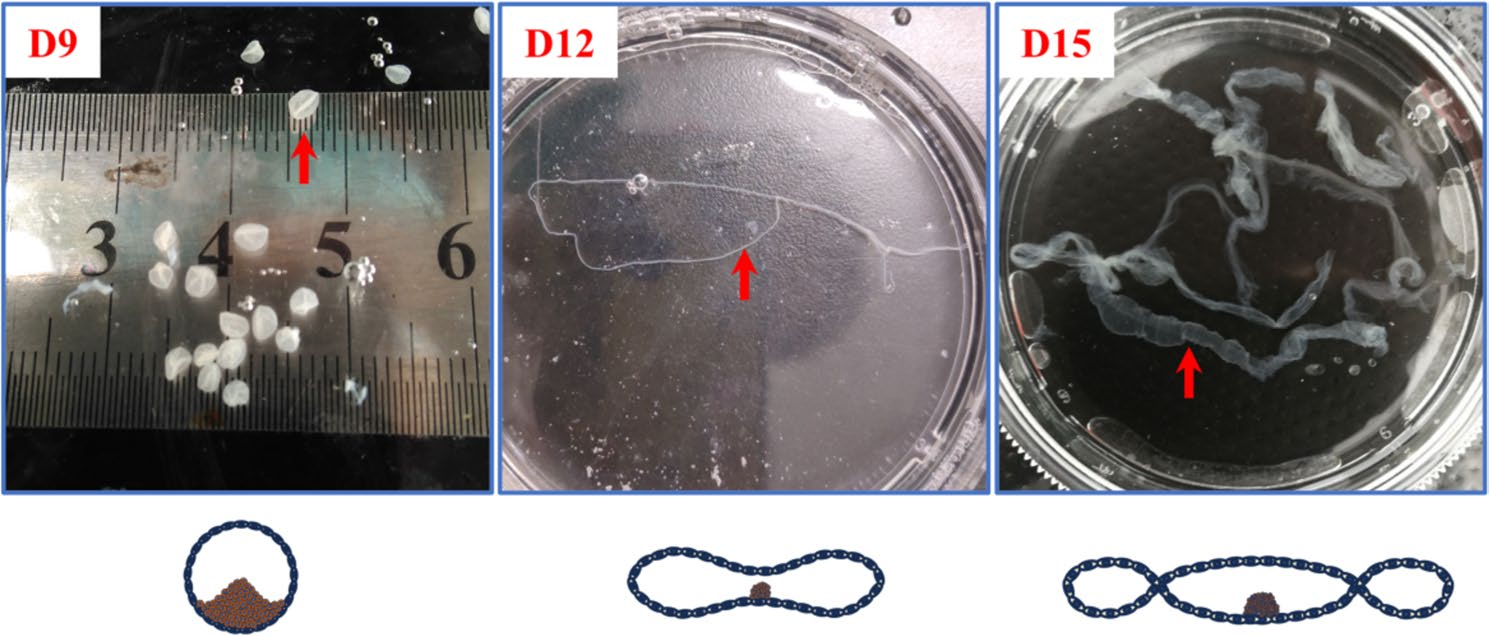
Morphology of porcine embryos on gestation D9, D12, D15

A significant restructuring of the porcine embryo’s morphology occurs throughout the peri-implantation phase. An examination of the morphology of early gestation embryos revealed that the porcine embryo adopts a spherical shape on D9 of gestation, with an approximate diameter of 3 mm, a thin filamentous shape by D12 of gestation, and a longer, thicker filamentous shape by D15 of gestation (Fig. 1).

### Dynamic changes of circRNAs transcripts in porcine embryos

**Fig. 2:**
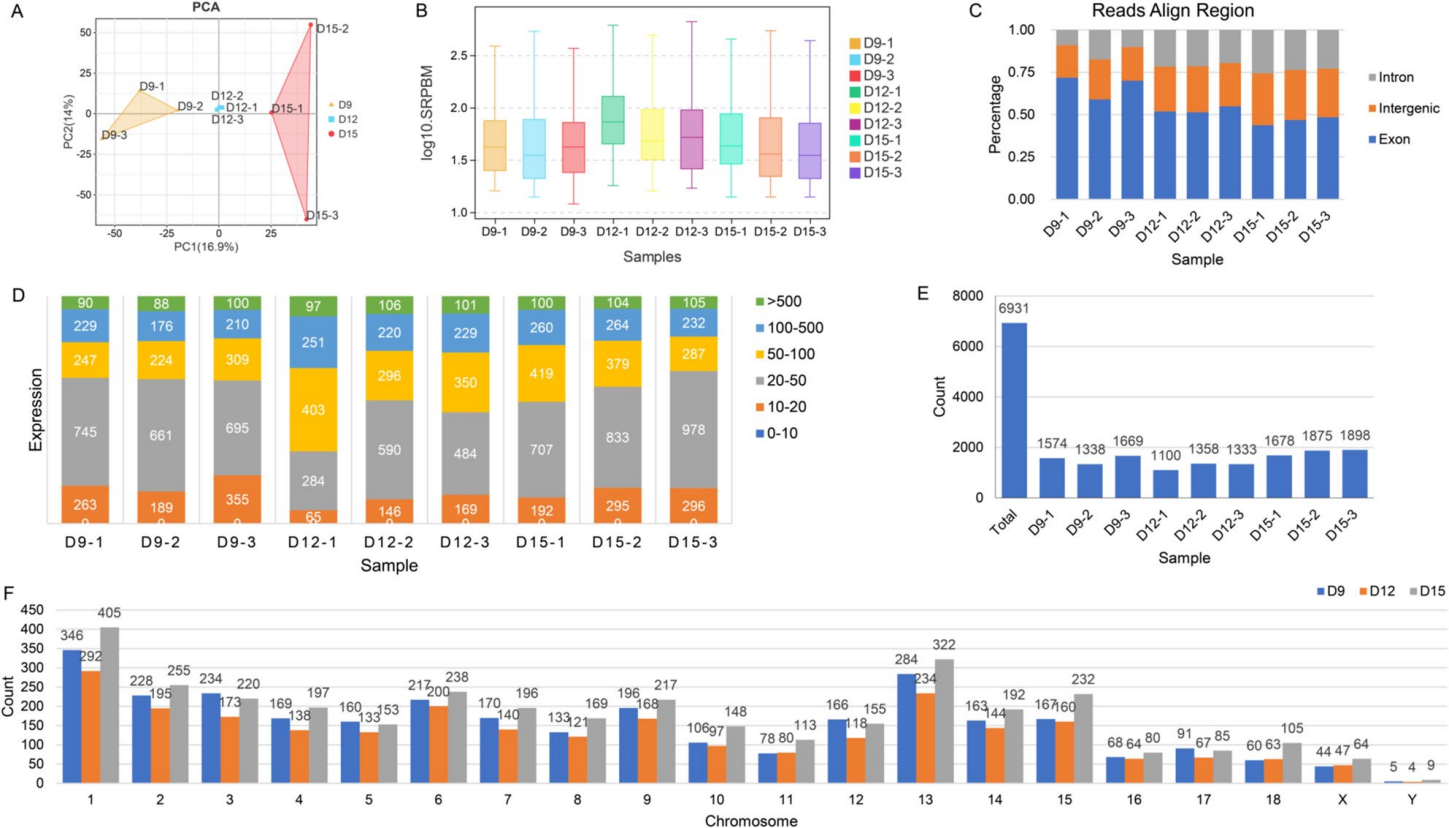
Expression characteristics of circRNA in porcine embryos during early pregnancy. **A** PCA-2D based on SRPBM values characterized the expression distribution of circRNAs in porcine embryos at D9, D12, and D15. **B** Box plot comparing circRNA expression levels across all samples, as quantified by SRPBM. **C** Reads comparison reference region statistics. **D** Number and proportion of circRNAs across SRPBM-based expression ranges in each sample. **E** Number of identified circRNAs in D9, D12, and D15 embryos. **F** Number of identified circRNAs on each chromosome

This study aimed to profile the expression dynamics of circRNAs throughout early gestation in porcine embryos. The quality control of the raw reads revealed that the Q20 value exceeded 96%, while the Q30 value surpassed 90% (Supplementary Material 3). Quantification was performed on the clean reads obtained from embryo sequencing, resulting in a total of 847,925,310 Paired-end (PE) reads (PE150) generated from nine pigs, generated using the Illumina paired RNA-seq method (Table 1); this equates to a total of 126.41 GB of data. Subsequently, the data were subjected to comparison and analysis, with the result that over 94.8% of the clean reads matched the genome. Subsequently, principal component analysis (PCA) was applied to analyze the circRNA expression profiles, which revealed that the expression data of embryos at three distinct gestational stages (D9, D12, and D15) could be significantly differentiated (Fig. 2A). Additionally, the expression profiles of the samples were examined, and the findings indicated that the expression levels were relatively high on D12 of gestation and relatively low on D9 and D15 of gestation (Fig. 2B). Furthermore, circRNAs were predominantly distributed in the exon region, with approximately 60% of circRNAs on D9 of gestation and 50% of circRNAs on D12 and D15 of gestation, respectively, located in this region (Fig. 2C). The expression levels of circRNAs in the samples were predominantly distributed within the range of 20–50 (Fig. 2D). A total of 6,931 circRNAs were identified across the three sample types (Fig. 2E, Supplementary Material 4.), with the highest number of circRNAs detected on D15 of gestation. The chromosome distribution analysis revealed that most circRNAs were located on chromosomes 1, 2, 3, 6, 9, 13, and 15 (Fig. 2F).

**Table 1:** Clean reads mapped to the reference genome

Samples	Total reads	Total mapped	Multiple mapped	Uniquely mapped	Read1	Read2	Reads map to ‘+’	Reads map to ‘-’	Reads mapped in proper pairs
D9-1	78,685,802	74,891,341 (95.18%)	2,404,136 (3.06%)	72,487,205 (92.12%)	36,957,432	35,529,773	36,155,620	36,331,585	69,389,568
D9-2	99,576,740	95,517,811 (95.92%)	2,376,662 (2.39%)	93,141,149 (93.54%)	47,017,337	46,123,812	46,508,729	46,632,420	89,905,738
D9-3	112,155,016	107,865,258 (96.18%)	3,458,736 (3.08%)	104,406,522 (93.09%)	52,762,490	51,644,032	52,120,200	52,286,322	100,980,318
D12-1	79,191,342	75,120,955 (94.86%)	1,469,099 (1.86%)	73,651,856 (93%)	37,311,165	36,340,691	36,809,347	36,842,509	70,518,156
D12-2	89,657,474	85,222,398 (95.05%)	1,903,152 (2.12%)	83,319,246 (92.93%)	42,091,018	41,228,228	41,645,616	41,673,630	80,067,602
D12-3	82,279,354	78,036,270 (94.84%)	1,964,616 (2.39%)	76,071,654 (92.46%)	38,468,898	37,602,756	38,023,913	38,047,741	72,971,888
D15-1	100,683,982	95,828,461 (95.18%)	1,467,021 (1.46%)	94,361,440 (93.72%)	47,658,226	46,703,214	47,130,685	47,230,755	90,754,244
D15-2	99,272,264	94,786,153 (95.48%)	1,622,227 (1.63%)	93,163,926 (93.85%)	47,091,025	46,072,901	46,554,018	46,609,908	89,788,582
D15-3	101,274,606	96,509,366 (95.29%)	1,673,377 (1.65%)	94,835,989 (93.64%)	47,916,930	46,919,059	47,391,372	47,444,617	91,183,436

(1) Total reads: number of clean reads. (2) Total mapped: clean reads aligned to the reference genome. (3) Multiple mapped: reads mapped to more than one genomic locus. (4) Uniquely mapped: reads aligned to a single locus. (5) Reads map to ‘+’, Reads map to ‘-’: reads assigned to the forward or reverse genomic strand. (6) Reads mapped in proper pairs: paired-end reads aligned in correct orientation and expected insert size

### High-abundance and DEcircRNAs in porcine embryos

**Fig. 3:**
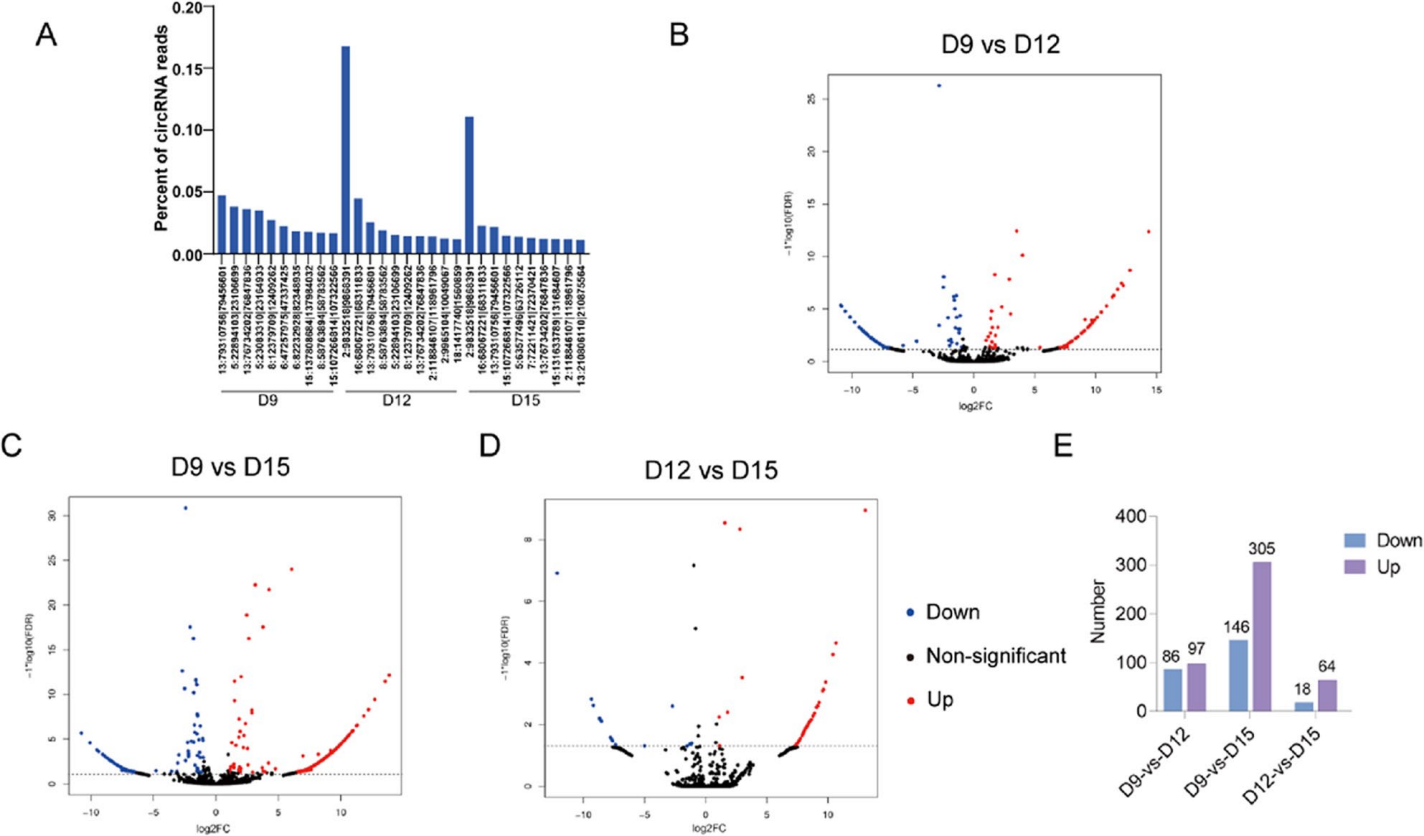
Overview of circRNA expression profiles and differential expression analysis in porcine embryos at different developmental stages. **A** Relative abundance of the top 10 circRNAs at D9, D12, and D15. circRNAs are ranked independently within each developmental stage, and the order does not imply matching of circRNAs across stages. Volcano plots of DEcircRNAs in D9 vs. D12 (**B**), D9 vs. D15 (**C**), and D12 vs. D15 (**D**). The X-axis represents the fold change (log₂), and the Y-axis represents the FDR value (− log10). The color of each point indicates the direction of expression change between groups: blue for down-regulated, red for up-regulated, and black for non-significant circRNAs. DEcircRNAs were defined by the thresholds of FDR < 0.05 and |log2FoldChange| > 1. **E** Number of up- and down-regulated circRNAs for each pair of groups

To characterise circRNA expression profiles at different gestational stages (D9, D12, and D15), we analysed the relative abundance of circRNAs within each stage independently. Figure 3A shows the ten most abundant circRNAs ranked separately for each developmental stage, rather than implying direct correspondence across stages. At D9, circRNA13:79310756|79,456,601 showed the highest relative abundance; at D12 and D15, circRNA2:9832518|9,868,391 was the most highly expressed, reaching 11.07% at D15. The DEcircRNAs were screened in each sample pair (D9 vs. D12, D9 vs. D15, D12 vs. D15), identifying 183, 634, and 82 DEcircRNAs, respectively (Fig. 3B-E, Supplementary Material 5–7).

### Hierarchical clustering and identification of hub circRNAs

**Fig. 4:**
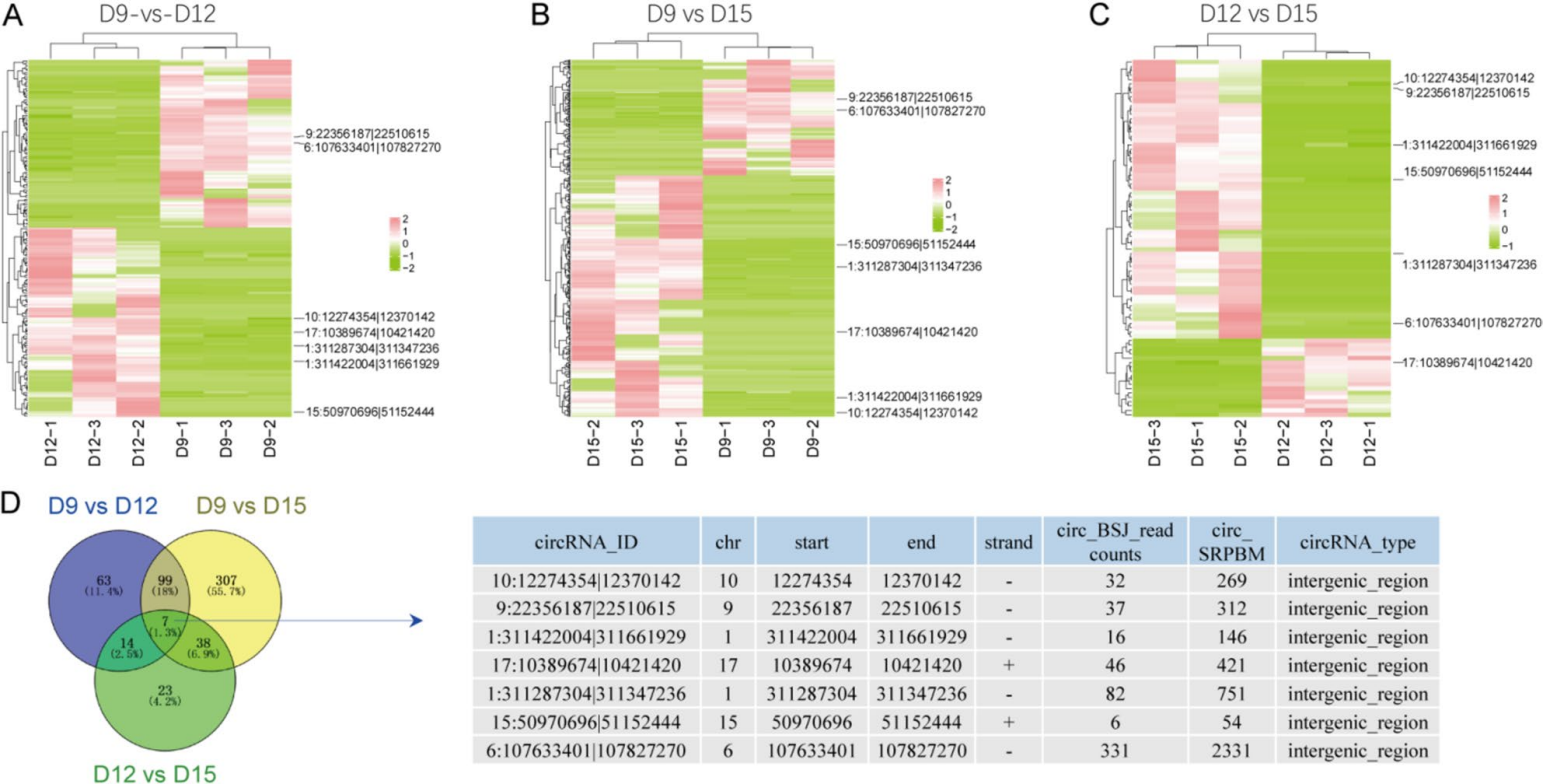
DEcircRNAs were analyzed across developmental stages to reveal expression patterns and overlaps. Hierarchical clustering of DEcircRNAs between D9 and D12 **A**, D9 and D15 **B**, and D12 and D15 **C**. **D** Overlap of pairwise DEcircRNAs in D9, D12, and D15

Based on the DEcircRNAs identified in the pairwise comparisons (183 in D9 vs. D12, 634 in D9 vs. D15, and 82 in D12 vs. D15), hierarchical clustering analysis (HCA) was performed to visualise the global expression patterns of these DEcircRNAs across developmental stages. This analysis revealed consistent stage-dependent expression trends and enabled the subsequent identification of a subset of hub circRNAs with the most prominent expression dynamics (Fig. 4A–C). To further identify Hub circRNAs, the overlap of DEcircRNAs among the three pairwise comparisons was examined, and a Venn diagram analysis revealed seven hub circRNAs shared across the comparisons. Detailed information on these circRNAs is provided in the table shown in Fig. 4D.

### Functional enrichment analysis of DEcircRNAs

**Fig. 5:**
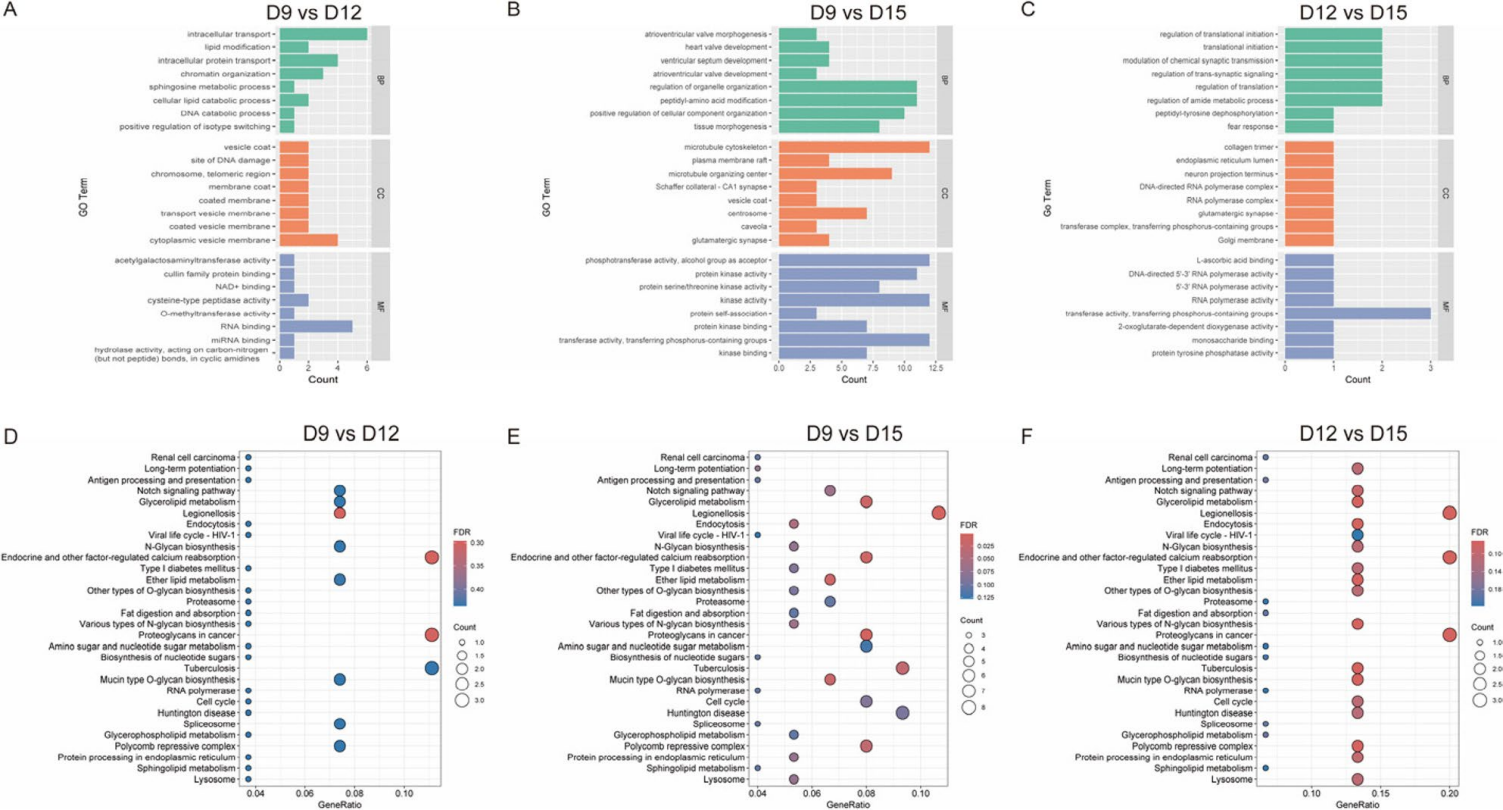
Functional enrichment analysis of DEcircRNAs. GO-based functional categorisation bar graph derived from host genes of DEcircRNAs for D9 vs. D12 **A**, D9 vs. D15 **B**, and D12 vs. D15 **C**. KEGG enrichment scatter plots of host genes of DEcircRNAs for D9 vs. D12 **D**, D9 vs. D15 **E**, and D12 vs. D15 **F**

In order to ascertain the potential role of the identified DEcircRNAs during embryonic development, GO functional annotation and KEGG pathway analysis were performed on the host genes of DEcircRNAs, showing that these circRNAs were predominantly enriched in biological processes, including atrioventricular valve morphogenesis, tissue morphogenesis, chromatin organization, regulation of organelle organization, peptidyl–amino acid modification, and regulation of translational initiation. The KEGG pathway enrichment analysis revealed a notable enrichment in the Cell growth and death, Transcription, Chromosome, Signal transduction, and Nervous system among the D9 vs. D12 group. Significant enrichment was observed in the TGF-β, Cell cycle, ErbB, and Adherens junction signalling pathways for the D9 vs. D15 group. Significant enrichment was observed in the TGF-β, ErbB, mTOR, AMPK, and Insulin signalling pathways for the D12 vs. D15 group (Fig. 5); both pathways were known to play essential roles in embryonic development.

### Hub mRNA analysis and ceRNA network construction

**Fig. 6:**
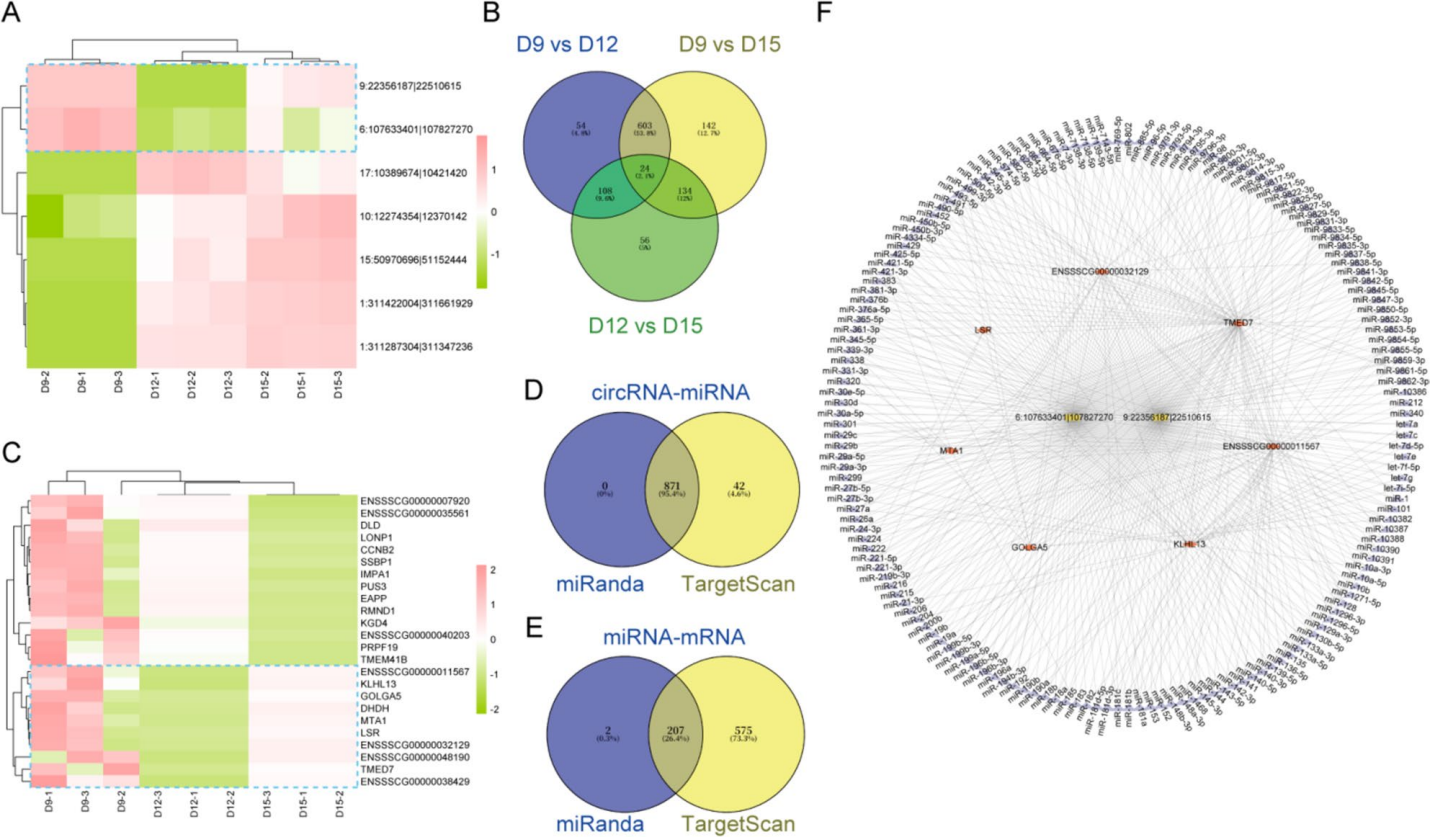
Analysis of ceRNA interactions. **A** HCA of hub circRNAs was performed across gestational D9, D12, and D15. **B** Overlap of pairwise DEmRNAs in D9, D12, and D15. **C** HCA of hub mRNAs was performed across gestational D9, D12, and D15. **D** Venn diagram showing the overlap of predicted circRNA–miRNA interactions. miRNAs potentially interacting with the two circRNAs were predicted using miRanda (blue) and TargetScan (yellow). Both tools predicted a total of 871 interactions (95.4%), while 42 interactions (4.6%) were uniquely predicted by TargetScan and none were uniquely predicted by miRanda. **E** Venn diagram showing the overlap of predicted miRNA–mRNA interactions. miRNAs potentially interacting with the seven mRNAs were predicted using miRanda (blue) and TargetScan (yellow). Both tools predicted a total of 207 interactions (26.4%), while 575 interactions (73.3%) were uniquely predicted by TargetScan, and two interactions (0.3%) were uniquely predicted by miRanda. (F) ceRNA interaction network. Red diamonds represent mRNAs, yellow rectangles represent circRNAs, and purple triangles represent miRNAs. Edges indicate predicted interaction relationships

Given that the host genes of DEcircRNAs were significantly enriched in multiple developmental- and signalling-related pathways, we next sought to explore the potential regulatory mechanisms underlying these associations. In particular, we investigated whether circRNAs might influence embryonic development through circRNA–miRNA–mRNA regulatory axes. To this end, we further characterize the expression of the seven hub circRNAs, HCA was performed, revealing two distinct temporal expression patterns across D9, D12, and D15 of gestation: high–low–medium and low–high–high (Fig. 6A). Based on the mRNA sequencing data obtained in this study, Differential expression analysis was conducted for all pairwise comparisons between developmental stages (D9 vs. D12, D9 vs. D15, and D12 vs. D15) to identify differentially expressed mRNAs (DEmRNAs). A total of 789, 903, and 322 DEmRNAs were detected in these comparisons, respectively (Supplementary Material 8). A Venn diagram of the DEmRNAs from the three comparisons identified 24 hub mRNAs (Fig. 6B). Hierarchical clustering of these hub mRNAs revealed two predominant temporal expression patterns across D9, D12, and D15 of gestation: high–medium–low and high–low–medium (Fig. 6C). Considering that circRNAs can function as molecular sponges for miRNAs and thereby regulate mRNA expression, the expression trends of circRNAs and their target mRNAs are expected to be consistent. In this study, both hub circRNAs and hub mRNAs exhibited a comparable high–medium–low expression pattern across the developmental stages. Based on this consistent trend, two circRNAs and ten hub mRNAs were selected for subsequent analysis. However, because ENSSSCG00000038429, ENSSSCG00000048190, and DHDH lack annotated 3′UTR regions, only two circRNAs and seven mRNAs were ultimately used for subsequent miRNA target prediction analysis. A total of 871 circRNA–miRNA and 207 miRNA–mRNA targeted interactions were identified (Fig. 6D–E). By integrating the miRNAs involved in both types of interactions, 166 miRNAs that potentially target both circRNAs and mRNAs were identified. These interactions were subsequently used to construct the ceRNA regulatory network (Fig. 6F, Supplementary Materials 9–10).

### Validation of circRNA junctions and expression by RT-qPCR

**Fig. 7:**
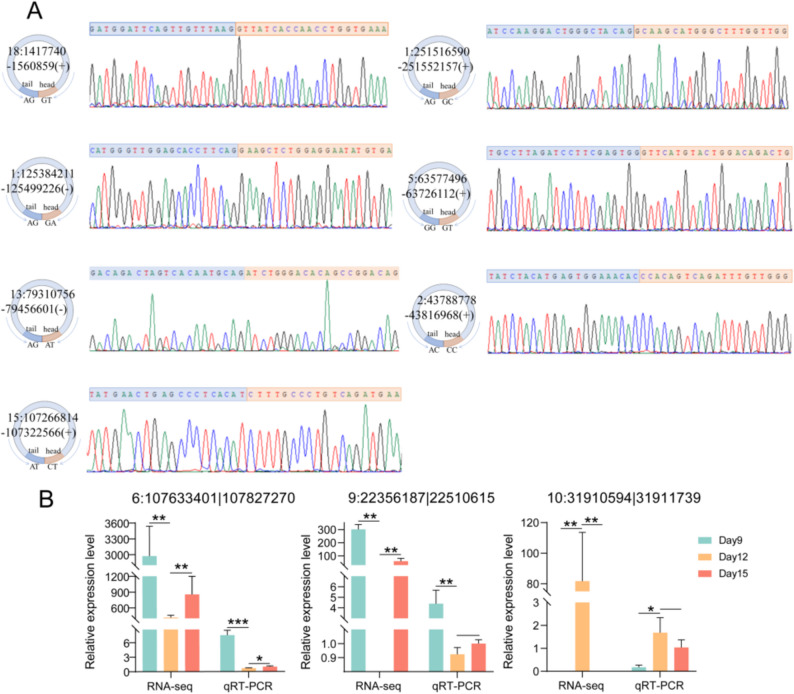
Experimental validation of circRNAs. **A** Verification of circRNA BSJ by Sanger sequencing. **B** RT-qPCR was performed to verify the DEcircRNAs between D9, D12, and D15. Results are expressed as mean ± standard deviation (SD), **represents *p-value* < 0.01, *represents *p-value* < 0.05

To evaluate the circular structure of circRNAs, we first randomly selected seven circRNAs (18:1417740–1560859, 1:125384211–125499226, 13:79310756–79456601, 15: 107266814–107322566, 1: 251516590–251552157, 5: 63577496–63726112, 2: 43788778–43816968), and after designing divergent primers, the BSJ was validated by PCR amplification and Sanger sequencing of cDNA, and as a result, the seven circRNAs were found to be circular (Fig. 7A). To validate the accuracy of the RNA-seq results, two key DEcircRNAs (6:107633401|107827270 and 9:22356187|22510615) and one randomly selected DEcircRNA (10:31910594|31911739) were subjected to RT-qPCR analysis in embryos at gestation D9, D12, and D15 to examine their expression levels. The results showed that RT-qPCR was similar to RNA-seq, demonstrating the accuracy of the sequencing results (Fig. 7B).

## Discussion

Owing to their covalently closed circular structure, circRNAs are resistant to exonuclease-mediated degradation and therefore exhibit greater stability than mRNAs, although they are generally expressed at low levels; nevertheless, certain relatively abundant circRNAs can play regulatory roles in a variety of biological processes [26, 27]. In this study, we built a circRNA-mediated ceRNA interactions network. Consistent with the ceRNA network predicted in our analysis, previous studies have reported several circRNAs with experimentally supported regulatory functions. Derived from the GRN gene, Circ0001470 was found to stimulate EEC proliferation and cell cycle progression and to reduce apoptotic events. It served as a miRNA sponge for miR-140-3p, influencing PTGFR expression and ultimately triggering the MAPK pathway, thereby contributing to pregnancy maintenance [28]. Gao et al. showed that circKDM5B, derived from the KDM5B gene, was abundantly expressed in porcine embryos, and knockdown of circKDM5B reduces blastocyst formation as well as the total and trophectoderm cell numbers. At the same time, the addition of miR-128 inhibitors can rescue the negative effects of circKDM5B knockdown on blastocysts. CircKDM5B mediates miR-128 and its downstream genes, ultimately regulating early embryonic development in pigs [29]. Through RNA-seq, Cao et al. identified circRNAs in porcine oocytes and cumulus cells. Enrichment analysis suggested that the DEcircRNAs participate in pathways governing cell activity and oocyte maturation, with many functioning as miRNA sponges. Reduction of circARMC4 expression in oocytes markedly impaired first polar body extrusion and early embryo development [30]. Although we observed dynamic changes in circRNA expression during early porcine development, a limitation of this study is that the embryos were not sexed, which may have potentially confounded circRNA interpretation. Additionally, circRNA identification relied solely on CIRI2 without cross-tool validation, which may have introduced false positives.

In this study, differential expression analysis did not explicitly include sow or batch as covariates, and future work should consider incorporating these factors to account for potential random effects. In addition, DESeq2 assumes that most genes are not differentially expressed and that counts follow a negative binomial distribution; when transcriptomes from widely divergent developmental stages are compared, these assumptions may be partially violated, potentially affecting dispersion estimates and *p-value* accuracy. Based on these analyses, the present study identified a total of 6,931 circRNAs in nine embryonic samples, with exonic circRNAs being the predominant type. Among the DEcircRNAs, the intersection across the three pairwise comparisons yielded seven hub circRNAs that showed the most prominent expression dynamics. To further investigate the biological relevance of these stage-associated circRNAs, we performed functional enrichment analysis of the DEcircRNAs. These enriched biological processes—including atrioventricular valve morphogenesis [31], tissue morphogenesis [32], chromatin organization [33], regulation of organelle organization [34], peptidyl–amino acid modification [35], and regulation of translational initiation [36]—are fundamental to embryonic development, as they collectively govern early tissue patterning, organ formation, and genome regulatory dynamics during morphogenesis. Additionally, Enrichment analysis revealed multiple embryonic development-related pathways, including those associated with cell growth and death, transcriptional regulation, chromatin organization, as well as the TGF-β, ErbB, mTOR, and AMPK signalling pathways. During embryogenesis, programmed cell death (apoptosis) is essential for shaping tissues and organs by removing unnecessary or improperly specified cells, thereby ensuring proper morphological development [37]. Early embryonic development involves extensive chromatin reprogramming to activate the zygotic genome and establish lineage specification, underscoring the fundamental roles of chromatin remodeling and transcriptional regulation in embryo formation [38]. Dynamic changes in chromatin accessibility during embryogenesis coincide with the spatiotemporal activation of regulatory elements, which govern proper cell-fate determination and morphogenesis [39]. The TGF-β signalling pathway was pivotal in both vertebrates and invertebrates [40]. Li et al. demonstrated that Dppa2 and Dppa4 facilitate early embryonic development in mice and humans by regulating the Hippo, MAPK and TGF-β signalling, which acted as activators of zygotic genome transcription [41]. The ErbB signaling pathway regulates cell proliferation, migration, and body/embryonic axis patterning in early vertebrate embryos (such as frogs), serving as an indispensable key mechanism during gastrulation and morphogenesis [42]. mTOR signaling is critical for the transition from the 8-cell stage (morula) to the blastocyst stage. Its inhibition disrupts trophoblast differentiation, interferes with DNA methylation and transcriptional regulation, ultimately impairing embryonic development and implantation quality [43]. The addition of genistein to chicken feed has been demonstrated to activate the AMPK pathway, thereby significantly improving the development and metabolism of chick embryos [44]. Since circRNAs may exert biological functions independent of their host genes, the enrichment analysis of host genes provides only an indirect indication of possible biological processes and should be interpreted with caution.

A total of 166 miRNAs were identified in the course of this study. Some miRNAs have been demonstrated to play a role in embryonic development and implantation. This study demonstrates that miR-181 impairs embryo implantation by directly targeting and suppressing LIF expression, under the regulation of the transcription factor Emx2, revealing a critical role of the Emx2–miR-181–LIF axis in embryo implantation [45]. This study reveals that the let-7 miRNA family suppresses the self-renewal of Dgcr8-deficient embryonic stem cells by downregulating self-renewal genes, while ESCC miRNAs antagonize this effect, thereby maintaining the balance between stem cell self-renewal and differentiation [46]. It has been reported that miR-21 regulates embryo development by modulating oocyte cytoplasmic maturation and cumulus expansion through the regulation of key genes such as Bmpr2, Ptx3, Cdk2ap1, and Oct4 in cumulus cells [47].

The regulatory role of circRNAs is primarily achieved through sponging miRNAs, which diminishes their suppressive action on downstream mRNA targets [26, 48, 49]. To explore the potential functional consequences of DEcircRNA-mediated miRNA regulation, we examined downstream targets highlighted in our predicted network. Before focusing on specific candidate genes, it should be noted that the number of differentially expressed mRNAs identified in the present study differs substantially from that reported in our previous work [22]. Although both studies analyzed the same RNA-seq datasets, the markedly different numbers of differentially expressed genes primarily reflect substantial methodological differences. The earlier study was conducted using the older pig reference genome Sscrofa10.2 with an early Ensembl annotation, which is known to contain fragmented gene models and redundant transcript annotations, thereby inflating the number of identified DEGs. In contrast, the present study re-analyzed the data using the updated Sscrofa11.1 genome assembly and Ensembl release 115, enabling improved consolidation of gene models and a clearer distinction between mRNAs and non-coding transcripts. In addition, the two studies differed in expression quantification and differential analysis strategies. The earlier analysis relied on FPKM-based expression estimates and DESeq (v1.18.0), which is more sensitive to low-expression variability, whereas the current study employed DESeq2 with raw count–based modeling, independent filtering, and variance shrinkage, resulting in more stringent control of false positives. Furthermore, the present study specifically focused on differentially expressed mRNAs and applied an additional intersection-based filtering to identify hub mRNAs consistently altered across developmental stages. Collectively, these methodological refinements produced a more conservative yet biologically robust set of DEmRNAs, accounting for the substantial reduction in the number of differentially expressed genes compared with the earlier report.

With this clarification in mind, we next focused on development-related genes within the network, including development-related genes such as MTA1 and LSR. Recent studies have highlighted the multifaceted roles of MTA1 in embryonic and placental development. MTA1 has been shown to directly interact with the transcription factor TFCP2L1 to maintain embryonic stem cell self-renewal and suppress endodermal differentiation, suggesting that MTA1 acts as a critical co-regulator in the pluripotency network [50]. In the placenta, dexamethasone-induced intrauterine growth restriction is accompanied by decreased MTA1 and increased MTA3 expression, while progesterone treatment reverses these changes, indicating an essential role of MTA proteins in placental development and pregnancy maintenance [51]. Moreover, both MTA1 and its short isoform MTA1s are up-regulated during late gestation and colocalize with estrogen receptors ERα and ERβ in placental cells, implying that MTA1 may modulate placental function and fetal development by regulating estrogen receptor signaling [52]. It has been reported that the LSR mediates lipoprotein clearance in the liver, and its deficiency results in impaired hepatic development and embryonic lethality, indicating that LSR is essential for liver formation and embryonic development [53].

Although the present study primarily focused on mRNAs and circRNAs, it is important to note that the re-analysis was performed using the updated Sscrofa11.1 genome assembly, in which lncRNA annotation has been substantially expanded and refined compared with earlier versions. Improved discrimination between mRNAs and lncRNAs under the updated annotation framework may influence read assignment and transcript quantification, thereby indirectly affecting the identification of differentially expressed mRNAs. During the peri-implantation period, lncRNAs are increasingly recognized as key regulators of gene expression through cis- and trans-acting mechanisms, including modulation of transcriptional activity and interaction with mRNAs. Although lncRNAs were not analyzed as a primary focus in this study, the mRNA expression dynamics observed here should be interpreted within the broader context of coordinated regulation among multiple RNA species. Future integrative analyses incorporating lncRNAs may further elucidate the complex regulatory networks governing peri-implantation development. lncRNAs may act as regulatory layers that modulate the expression of peri-implantation–related mRNAs, contributing to the fine-tuning of transcriptional programs required for successful implantation.

While this study provides valuable insights by profiling circRNAs and constructing a ceRNA network, the predicted regulatory interactions were not experimentally validated. As such, the proposed functions of circRNAs remain speculative. Future work should include functional assays to confirm the roles of specific circRNAs in early pregnancy and investigate circRNA–miRNA–mRNA crosstalk at the network level to better understand the regulatory mechanisms underlying embryonic development.

## Conclusions

In conclusion, the identification of circRNAs in porcine embryonic tissues during early pregnancy, revealed through RNA-seq analysis on D9, D12, and D15, underscores their potential regulatory roles in reproductive biology. The study identified DEcircRNAs, their associated biological processes, downstream targeted miRNAs, and hub mRNAs within the ceRNA network, and further validated these findings through experimental methods. Pathway enrichment analysis suggests a link between specific circRNAs and embryonic development. Further studies should clarify the molecular roles of these circRNAs to improve reproductive management in livestock. These findings may also serve as a basis for developing biomarkers and strategies to enhance reproductive efficiency in pigs.

## Supplementary Information

Supplementary Material 1.

Supplementary Material 2.

Supplementary Material 3.

Supplementary Material 4.

Supplementary Material 5.

Supplementary Material 6.

Supplementary Material 7.

Supplementary Material 8.

Supplementary Material 9.

Supplementary Material 10.

## Data Availability

The RNA-Seq dataset analysed in this study has been previously published and is publicly available in the NCBI SRA under the BioProject accession number PRJNA646603. No new sequencing data were generated in this study. All analysis results produced here are included in this article and its Supplementary Information files.

## Abbreviations

D: Day
circRNAs: Circular RNAs
ncRNA: Non-coding RNA
DEcircRNAs: Differentially expressed circRNAs
DEmRNAs ceRNA: Differentially expressed mRNAs competing endogenous RNA
PCR: Polymerase chain reaction
RT-qPCR: Real-time quantitative PCR
miRNAs: MicroRNAs
PE: Paired-end
BSJ: Back-Spliced Junction
SRPBM: Spliced reads per billion mapping
FDR: Adjusted p-value
FPKM: Fragments Per Kilobase of exon model per Million mapped fragments
GO: Gene Ontology
KEGG: Kyoto Encyclopedia of Genes and Genomes
EIciRNAs: Exon–intron circRNAs
3’ UTR: 3’-untranslated regions
PCA: Principal component analysis
HCA: Hierarchical clustering analysis
SD: Standard deviation
EECs: Endometrial epithelial cells
EVs: Extracellular vesicles
